# Inhangapi Virus: Genome Sequencing of a Brazilian Ungrouped Rhabdovirus Isolated in the Amazon Region

**DOI:** 10.1128/genomeA.01525-15

**Published:** 2016-01-21

**Authors:** Ana Lucia M. Wanzeller, Márcio R. T. Nunes, Fernando N. Tavares, Walber V. M. Pinto, Edivaldo C. Júnior, Clayton P. S. de Lima, Layanna F. de Oliveira, João Lídio S. G. V. Júnior, Jedson F. Cardoso, Pedro F. C. Vasconcelos

**Affiliations:** a Department of Virology, Evandro Chagas Institute, Ministry of Health, Ananindeua, Brazil; b Center for Technological Innovation, Evandro Chagas Institute, Ministry of Health, Ananindeua, Brazil; c Department of the Environment, Evandro Chagas Institute, Ministry of Health, Ananindeua, Brazil; d Department of Arbovirology and Hemorrhagic Fevers, Evandro Chagas Institute, Ministry of Health, Ananindeua, Brazil

## Abstract

We report here nearly complete genome sequence of Inhangapi virus (INHV) strain BEAR177325, which was isolated from a pool of sandflies (*Lutzomyia flaviscutellata*) in the Utinga neighborhood, Belém (01º28´S 48°27′W), State of Pará, Brazil, in 1969. The genome of this virus showed similarity with members belonging to the family *Rhabdoviridae*.

## GENOME ANNOUNCEMENT

The family *Rhabdoviridae* belongs to the order *Mononegavirales* and currently is composed of 67 members distributed in 11 genera and an unassigned genus (1). Rhabdoviruses are enveloped, have a helical nucleocapsid bullet shape, and are approximately 100 to 430 nm long and 45 to 100 nm in diameter. Rhabdoviruses have single-stranded, negative-sense, and nonsegmented RNA. The rhabdoviruses present five structural genes (N, P, L, M, and G) (2), accessory genes, and intergenic regions (3, 4). Viruses belonging to this family infect a variety of animals (vertebrates and invertebrates) and plants (5, 6), and transmission often requires arthropod vectors (mosquitoes, sandflies, and ticks) (1). Sigma virus (type species *Drosophila melanogaster* sigma virus [DmelSV]) is an exception, because it is transmitted vertically among parent flies (i.e., through both eggs and sperm) (5).

The Inhangapi virus (INHV) (BEAR177325) was isolated from a pool of sandflies (*Lutzomyia flaviscutellata*) in the Utinga neighborhood, Belém (01º28´S and 48°27′W) (7), State of Pará, Brazil, in 1969. The nearly complete genome of INHV is around 12,020 bp and contains the five typical rhabdovirus genes N, P, M, G, and L.

Classic tests, such as complement fixation and neutralization tests, and transmission electron microscopy suggest that INHV is a member of the family *Rhabdoviridae* (7).

INHV particles were precipitated using polyethylene glycol 8000 (PEG 8000) centrifugation, as previously described (4), and the supernatant was treated with DNase and RNase (Ambion) for host contaminant removing. RNA extraction was followed by full-length genome sequencing using two different next-generation sequencing platforms: GS FLX 454 (Roche Life Science) and Ion Torrent (Life Technologies). Regardless of the sequencing platform used, the method for obtaining the genome basically involved the following steps: RNA fragmentation, library preparation (cDNA), emulsion PCR, and sequencing, as previously described (8, 9). The sequencing steps were carried out at the genomic core of the Center for Technological Innovation, Evandro Chagas Institute, Brazilian Ministry of Health, Ananindeua, Brazil.

The genome was obtained employing a *de novo* hybrid assembly strategy using both Ion Torrent and GS FLX 454 reads with the software Mira 4.0. Visual inspection was performed with the software Geneious version 6.1.4. The total genome recovered was 12,020 nucleotides (nt) in length, with a mean coverage of 383-fold. The five main genes 3′-N−P-M-G-L-5′ were recognized, as well as the putative accessory gene (pAG1) between the G and L genes. This is the report of the nearly complete genome sequence for the original passage to INHV, an ungrouped Brazilian rhabdovirus.

### Nucleotide sequence accession number. 

The nearly complete genome sequence has been deposited in GenBank under the accession number KR604694.

## References

[1] Dietzgen RG, Calisher CH, Kurath G, Kuzman IV, Rodriguez LL, Stone DM, Tesh RB, Tordo N, Walker PJ, Wetzel T, Whitfield AE Rhabdoviridae, p 654–681. In King AMQ, Adams MJ, Carstens EB, Lefkowitz EJ (ed), Virus taxonomy, Ninth Report of the International Committee on Taxonomy of Viruses. Elsevier, San Diego, CA.

[2] Tordo N, Benmansour A, Calisher C, Dietzgen RG, Fang RX, Jackson AO, Kurath G, Nadin-Davis S, Tesh RB, Walker PJ Family *Rhabdoviridae*, p 623–644. In Fauquet CM, Mayo MA, Maniloff J, Desselberger U, Ball LA (ed), Virus taxonomy. Eight Report of the International Committee on Taxonomy of viruses. Elsevier, San Diego, CA.

[3] Walker PJ, Firth C, Widen SG, Blasdell KR, Guzman H, Wood TG, Paradkar PN, Holmes EC, Tesh RB, Vasilakis N 2015 Evolution of genome size and complexity in the *Rhabdoviridae*. PLoS Pathog 11:e1004664. doi:10.1371/journal.ppat.1004664. 25679389 PMC4334499

[4] Wanzeller ALM, Martins LC, Diniz Júnior JAP, Medeiros DBA, Cardoso JF, da Silva DEA, Oliveira LF, Vasconcelos JM, Nunes MRT, Vianez Júnior JLSG, Vasconcelos PFC, Virus X 2014 A hitherto undescribed virus within the family *Rhabdoviridae* isolated in the Brazilian Amazon region. Genome Announc 2(3):e00454-14. doi:10.1128/genomeA.00454-14. 24948755 PMC4064021

[5] Lyles DS, Kuzmin IV, Rupprecht CE 2013 Rhabdoviridae, p 885–922. *In* Knipe DM, Howley PM (ed), Fields virology, 6th ed. Lippincott Williams & Wilkins, Philadelphia, PA.

[6] Bourhy H, Cowley JA, Larrous F, Holmes EC, Walker PJ 2005 Phylogenetic relationships among rhabdoviruses inferred using the L polymerase gene. J Gen Virol 86:2849–2858. doi:10.1099/vir.0.81128-0. 16186241

[7] Karabatsos N 1985 International catalogue of arboviruses. American Society of Tropical Medicine and Hygiene, San Antonio, TX.

[8] Margulies M, Egholm M, Altman WE, Attiya S, Bader JS, Bemben LA, Berka J, Braverman MS, Chen YJ, Chen Z, Dewell SB, Du L, Fierro JM, Gomes XV, Godwin BC, He W, Helgesen S, Ho CH, Irzyk GP, Jando SC, Alenquer ML, Jarvie TP, Jirage KB, Kim JB, Knight JR, Lanza JR, Leamon JH, Lefkowitz SM, Lei M, Li J, Lohman KL, Lu H, Makhijani VB, McDade KE, McKenna MP, Myers EW, Nickerson E, Nobile JR, Plant R, Puc BP, Ronan MT, Roth GT, Sarkis GJ, Simons JF, Simpson JW, Srinivasan M, Tartaro KR, Tomasz A, Vogt KA, Volkmer GA, et al. 2005 Genome sequencing in microfabricated high-density picolitre reactors. Nature 437:376–380. 16056220 10.1038/nature03959 PMC1464427

[9] Rothberg JM, Hinz W Rearick TM, Schultz J, Mileski W, Davey M, Leamon JH, Johnson K, Milgrew MJ, Edwards M, Hoon J, Simons JF, Marran D, Myers JW, Davidson JF, Branting A, Nobile JR, Puc BP, Light D, Clark TA, Huber M, Branciforte JT, Stoner IB, Cawley SE, Lyons M 2011 An integrated semiconductor device enabling non-optical genome sequencing. Nature 475:348–352 doi:10.1038/nature10242. 21776081

